# EditABLE: A Simple Web Application for Designing Genome Editing Experiments

**DOI:** 10.21203/rs.3.rs-4775705/v1

**Published:** 2024-08-16

**Authors:** Demetrios S. Maxim, David Wei Wu, Najani Shanee Johnson, Vivek Charu, Jennefer N. Carter, Shuchi Anand, George M. Church, Vivek Bhalla

## Abstract

CRISPR–Cas genome editing is transformative; however, there is no simple tool available for determining the optimal genome editing technology to create specific mutations for experimentation or to correct mutations as a curative therapy for specific diseases. We developed editABLE, an online resource (editable-app.stanford.edu) to provide computationally validated CRISPR editors and guide RNAs based on user provided sequence data. We demonstrate the utility of editABLE by applying it to one of the most common monogenic disorders, autosomal dominant polycystic kidney disease (ADPKD), identifying specific editing tools across the landscape of ADPKD mutations.

## BACKGROUND

CRISPR–Cas is a highly versatile genome editing system that has fundamentally altered several fields of research, including gene therapy (1). CRISPR–Cas initiates double-stranded breaks in DNA and relies on cellular repair to occur via one of two pathways: nonhomologous end joining or homology-directed repair in the presence of a donor template. However, homology-directed repair is very inefficient (< 1% in most cell types) (2),(3).

Base editing and prime editing are newer versions of the CRISPR–Cas system, which do not rely on double-stranded breaks, greatly expanding the percentage of mutations that can be efficiently corrected with genome editing (4),(5). Base editing corrects transition mutations (A->G, T->C, C->T, G->A) and certain transversion mutations (C->G, G->C) and is several orders of magnitude more efficient than homology-directed repair (50–75% vs. 0.1–10%) (4),(6). Prime editing can correct all 12 point mutations as well as insertions and deletions up to 80 base pairs (7),(8). Other technologies similar to prime editing have also been described that allow for larger insertions and deletions such as integrases and twin prime editing (9),(10). Prime editing and integrases are promising, but for correcting single transition mutations with an available PAM, base editing is two- to tenfold more efficient (7).

Approximately > 95% of all human pathogenic mutations can now, in theory, be corrected via these four editing technologies (base editing, prime editing, twin prime editing, and integrases). However, new versions of these technologies are published frequently. This changing landscape makes it challenging for researchers to ensure that they are using the optimal, most efficient editing methods in their experiments. In addition, there is no resource available that combines this information and guide RNA design into a single online tool.

In this study, we present editABLE, a publicly available tool for designing genome editing experiments and potential therapies. We illustrate the use of editABLE in autosomal dominant polycystic kidney disease (ADPKD), one of the world’s most common monogenic disorders, and conduct a systematic analysis of the ADPKD mutational landscape.

## RESULTS AND DISCUSSION

We developed the editABLE algorithm in Python **(Supplementary Figs. 1 & 2)** and applied this algorithm to mutation data from the Mayo Clinic ADPKD Mutation Database to identify the percentage of ADPKD patients carrying mutations correctable via base or prime editing. The editABLE algorithm takes two DNA sequences as inputs: the original sequence to be modified and the desired final sequence **(Supplementary Fig. 2)**. EditABLE outputs the optimal editing strategy and guide RNA(s) based on the algorithm. Bulk analysis is also supported. **Supplementary Table 1** provides an overview of the features available in the editABLE web application. Although some of these features are available in existing web tools, none of them are available in a single resource for simplistic use by scientists and clinicians new to genome editing.

Base editable mutations are divided into subgroups based on the specific type of base editor (adenine [ABE], cytosine [CBE], or transversion [CGBE]) appropriate for each mutation (4),(5),(11). For our ADPKD analysis, we defined base editable mutations as those with an NGN PAM sequence that positioned the substitution mutation 12–17 bp upstream of the PAM in the 5' direction, which was identified previously as the most efficient base editing window (6). We stratified the analyses between *PKD1* and *PKD2* mutations. Although NGG is the most efficient PAM for base editing with *Streptococcus pyogenes* Cas9, NGN shows comparable efficiency and allows for more flexible targeting (6).

For *PKD1*, 32%, 9%, and 2% of patients carry mutations that are correctable with ABE, CBE, and CGBE base editors respectively **(**Fig. 1A**).** Ninety-sex percent of these patients are potential candidates for therapy via base editing or prime editing. Our findings are similar for *PKD2*: 38%, 4%, and 1% of patients carry mutations that are correctable with ABE, CBE, or CGBE editors, respectively, and 93% of patients are potential candidates for therapy via base or prime editing **(Supplementary Fig. 3A)**. Most patients' pathogenic substitution mutations are amenable to base editing treatment, suggesting that NGN PAM availability in the *PKD1* and *PKD2* coding regions is not limiting **(**Fig. 1B, **Supplementary Fig. 3B).** This observation is consistent with other studies reporting high GC content (~ 85%) in both *PKD1* and *PKD2* (12),(13). The most common available PAM sites for *PKD1* and *PKD2* base editors are NGC (35% of sites for *PKD1* and 34% for *PKD2*) and NGG (31% of sites for *PKD1* and 28% of sites for *PKD2*) **(**Fig. 1C, **Supplementary Fig. 3C).**

We found that base editable and prime editable *PKD1* and *PKD2* mutations are scattered throughout the coding region and that there is no single hotspot mutation region **(**Fig. 1D, **Supplementary Fig. 3D).** Several hotspot mutation regions are noticeable and align with those reported previously by other investigators such as the 3' region of *PKD1*, the extracellular domain of *PKD2* (coding region base pairs: 672–1407), and the N-terminus, REJ, TOP, and PLAT domains in *PKD1* **(Supplementary Fig. 4)** (12),(14). Finally, among patients with variants in *PKD1* or *PKD2*, we conducted a mutational comparison between those who did and did not present with ADPKD and found that truncation mutations were the most common pathogenic variants **(**Fig. 1E, **Supplementary Fig. 3E)**. These findings also align with previous reports, including an analysis of 175,000 patients conducted by Chang and colleagues (15). Finally, we used editABLE to identify a candidate list of starting mutations for a base editing **(Supplementary Table 2)** or prime editing **(Supplementary Table 3)** clinical trial. This candidate list includes the most common *PKD1* truncation variants amenable to either base or prime editing with an NGN or NGG PAM site, respectively. *PKD1* truncation variants are ideal for early-stage clinical trials because they are associated with the most severe clinical phenotypes (12).

Next, we sought to validate the translatability of the editABLE algorithm in human renal epithelial cells isolated from ADPKD patients. We chose the *PKD1* nonsense truncation mutation, Q2556X (*PKD1*$^{Q2556X}$), because this variant is a common, severe mutation on our candidate list for establishing a clinical base editing proof-of-concept (**Supplementary Table 2)**. Based on the protocol in Fig. 2A, we observed *PKD1* correction efficiencies as high as 40% in unsorted cells **(**Fig. 2B**).** No significant differences were observed in editing efficiency between the target and nontarget strands, which supports 72 hours post transfection as a good timepoint for quantifying editing efficiency. We achieved 62% mCherry + GFP + via FACS and observed *PKD1* correction efficiencies as high as 66% in this sorted double-positive population **(**Fig. 2C, 2D**)**. These results were significantly different from those in untransfected cells **(**Fig. 2D**).** Compared with gRNA1 and gRNA1G, guide RNA2 was the optimal guide RNA in both the unsorted and the sorted populations and improved editing efficiency by 50%. However, gRNA1G did not significantly differ in performance compared with gRNA1 **(**Fig. 2B**)**. These results suggest that for this site, compared with other loci, the alteration of the 5’ gRNA nucleotide to guanine has no significant effect on editing efficiency (16). Due to the low efficiency of kidney gene delivery with existing technologies, *in vivo* genome editing with these guide RNAs is not yet feasible (data not shown) (17).

In this study, we present the development, implementation, and validation of editABLE, a novel computational, open-source tool of broad utility to genetics researchers and translational scientists for applying genome editing to experimental and potentially therapeutic applications. We have made this tool available publicly online (18) and will continue to maintain this tool as a service to the genome editing community. We show the broad utility of editABLE by providing use cases across different editing tools (base, prime editing), species (mouse, human), and diseases (ADPKD, sickle cell anemia, Hutchinson–Gilford progeria syndrome) (**Supplementary Note 2**). We also highlight the unique features of editABLE compared with existing tools **(Supplementary Table 1)**. EditABLE will facilitate the development of targeted gene editing therapies for multiple genetic diseases, building off existing gene editing clinical trials in humans (1),(19),(20).

One limitation of our *in vitro* validation experiments is that we did not test the full range of potential editing approaches. We used the ABEmax editor for our proof-of-concept experiments because it is the most well-characterized adenine base editor available, but further validation could test additional adenine base editors, such as ABE8e and ABE7.10 as well as various prime editors and prime editing guide RNAs. Future versions of the editABLE website may also incorporate computational predictions on off-target editing for each guide RNA or the addition of other CRISPR nucleases, such as Cas12a (21).

Although several existing tools exist for designing CRISPR–Cas experiments, none of these tools are easy to use for scientists who are completely new to genome editing. PnB Designer is the most similar tool to editABLE currently available and generates either prime- or base- editing guide RNAs based on single- or batch- sequence input data provided by the user (22). However, PnB Designer requires the user to select either base or prime editing as their desired editing strategy and does not provide an integrated algorithm to determine the optimal strategy based on the user provided sequences. PnB Designer also does not provide predictions of off-target or on-target editing. On the other hand, Benchling's Guide RNA Design Tool provides these off-target and on-target calculations but does not allow users to specify their desired edits; therefore, the Benchling tool is only useful for generating knockout mutations (23). EditABLE is designed for specific targeted mutations and combines the advantages of these prior tools into one algorithm that is simple to use. The genome editing field has grown exponentially in the last decade, and there is a clear need for a central open-source software resource that editABLE fills. EditABLE is also highly scalable, allowing for rapid integration of newer genome editing technologies as the field continues to advance.

### CONCLUSIONS

EditABLE is a novel open-source tool for the genome editing community that can be used to design guide RNAs for > 95% of human pathogenic variants. This study provides unique insights into the mutational landscape of one of the world's most common monogenic disorders and supports the development of future base and prime editing-based therapeutic approaches for ADPKD. Taken together, these results strongly support the further development of CRISPR base and prime editing as viable, potentially curative therapeutic approaches for the majority of patients with ADPKD.

### METHODS

## EditABLE Algorithm Implementation

We wrote a Python3 script based on the pseudocode from **Supplementary Fig. 1** to implement the editABLE algorithm. This Python script is a command line tool that requires the installation of the pandas, numpy, regex, and Bio packages (see **Supplementary Note 1**). To maximize the accessibility of editABLE to the broader research community, we developed a web portal interface implementation of editABLE (**Supplementary Fig. 2**). This website provides a graphical user interface that accepts user-provided sequence data and provides recommended editing strategies (18). EditABLE accepts single sequences input directly on the webpage or batch sequences uploaded in a CSV file. The editABLE web application also includes advanced settings that allow advanced users to select alternative PAM sequences and modify the base editing window. Example inputs and expected outputs for the editABLE website are shown for single-sequence inputs in **Supplementary Note 2** and for batch sequence inputs in **Supplementary Tables 4 & 5**. The editABLE web application uses the PrimeDesign algorithm to design prime editing guide RNAs (24).

## ADPKD Mutation Database Analysis

Several databases include variant information for *PKD1* and *PKD2*, including the Mayo Clinic PKD Mutation Database (2322 *PKD1* records), ClinVar (2847 *PKD1* records), gnomAD (5450 *PKD1* records), and the UK Biobank. These record counts are current as of June 13, 2023. We chose to focus our analysis on the Mayo Clinic Database because it is the only database that accounts for multiple pedigrees carrying the same mutation. This was critical because we sought to quantify which *PKD1* mutations can treat the largest number of patients with a single gene editor, which would not be possible without pedigree level data. The Mayo Clinic Database includes both pathogenic and nonpathogenic variants, so we excluded nonpathogenic variants from most analyses, except those in Fig. 1E and **Supplementary Fig. 3E**. First, we exported the Mayo Clinic data as a CSV file and imported it into Python via the pandas package. After correcting the notation for any mutations that were entered incorrectly, we applied the editABLE command line script to this organized pandas dataframe to generate the data shown in Fig. 1 and **Supplementary Fig. 3**. Unless otherwise indicated, we conducted patient-level analyses, so multiple unique pedigrees with a given variant were pooled together. We classified patients as amenable to base editing (ABE, CBE, or CGBE) if an individual mutation possessed an NGN PAM sequence that positioned the substitution mutation 12–17 bp upstream of the PAM in the 5' direction. One current limitation of our tool is that we defined base editable mutations by reversion to the reference sequence. For sites currently not designated as base editable, it may be possible to make a different base-editable substitution to revert to the original amino acid residue even if the nucleotide does not return to the reference sequence given the redundancy of the genetic code. However, alternate mutations may introduce issues with splicing if the point mutation is at an intron/exon boundary or with codon usage. We classified patients as amenable to prime editing if they harbored a nonbase-editable substitution mutation, a deletion mutation less than 44 bp, or an insertion mutation less than 80 bp. These specific numbers were used based on published data that indicate efficient prime editing with insertions as large as 44 bp and deletions as large as 80 bp (7). A deletion mutation must be corrected with an insertion, which is why the 44 bp and 80 bp numbers are flipped.

## Plasmid Preparations and Cloning

We sought to validate the guide RNA sequences produced by the editABLE algorithm *in vitro* via a dual plasmid cotransfection system. From our mutation candidate analysis in **Supplementary Table 2**, we identified the $PKD1^{7666C>T}$ mutation (protein: $PKD1^{Q2556X}$) as an ideal mutation for establishing base editing proof-of-concept because this mutation has a severe clinical phenotype and has been reported in several independent ADPKD families. We chose this specific mutation for our validation studies because there is an immortalized human renal epithelial cell line with the $PKD1^{Q2556X}$ mutation available from the American Type Culture Collection (Manassas, VA). The editABLE algorithm identified two possible guide RNAs for adenine base editing to correct the $PKD1^{Q2556X}$ mutation (gRNA1: CAGCTGGTCCTACACCACCA, gRNA2: GGTCCTACACCACCACGGCC both with NGG PAMs). We also tested a third guide RNA (gRNA1G: GAGCTGGTCCTACACCACCA), that was identical to gRNA1 except that the first base pair was changed from C to G for possible improvement of guide RNA transcription from the U6 promoter. We cloned these sequences into the mCherry-U6-empty plasmid (Addgene #140580, Watertown, MA). Integrated DNA Technologies (Coralville, IA) synthesized the guide RNA sequences. Briefly, we digested 10 µg of plasmid with BsmbI-v2 enzyme (New England Bio Labs [NEB], #R0739S, Ipswich, MA) at 55$^{o}$C for two hours and heat inactivated at 80$^{o}$C for 20 minutes. Next, we dephosphorylated the digested product by adding Antarctic Phosphatase (NEB #M0289S), incubating it at 37$^{o}$C for 15 minutes, and heat inactivating at 70$^{o}$C for five minutes. We phosphorylated and annealed the guide RNA oligonucleotides using T4 polynucleotide kinase (NEB #M0201S) at 37$^{o}$C for 30 minutes, and we ligated the annealed oligonucleotides with the digested mCherry-U6-empty plasmid using T4 DNA ligase (NEB #M0202S) for 2 hours at 25$^{o}$C. Lastly, we transformed the ligation product into 5-alpha competent E. coli (NEB, # C2987I) based on the manufacturer's instructions and sequence verified individual colonies resulting from the transformation. We isolated plasmid DNA from verified colonies using the ZymoPURE™ II Plasmid Purification Maxiprep Kit (Zymo Research #D4202, Irvine, CA). We repeated this Maxiprep protocol for the base editing plasmid used in our transfection (pCMV_ABEmax_P2A_GFP, Addgene: #112101).

## Cell Culture and Transfection

We used ADPKD cyst-derived human renal epithelial cells for all *in vitro* experiments, including control WT 9 – 7 cells ($PKD1^{Q2556X/+}$, ATCC #CRL-2830) and mutant WT 9–12 cells ($PKD1^{Q2556X/-}$, ATCC #CRL-2833).(25) We grew the cells in Dulbecco's modified Eagle Medium supplemented with 10% FBS and 1X antibiotic-antimycotic solution (Thermo Fisher #15240062, Waltham, MA). We maintained the cells through a weekly 1:10 split with 0.25% Trypsin-EDTA (Sigma-Aldrich #T4049, St. Louis, MO) but did not propagate for more than 20 passages. To transfect *PKD1* mutant cells with base editors, we used the Thermo Fisher Neon Electroporation device (Thermo Fisher: MPK5000) with a 1:3 mass ratio of mCherry-U6-guide RNA plasmid to pCMV_ABEmax_P2A_GFP plasmid (i.e. 500 ng mCherry plasmid and 1.5 µg GFP plasmid per well in a 6-well plate. We used 100,000–200,000 cells per reaction using the 10 µL Neon electroporation tips with the following settings (2 pulses, 1150 V voltage per pulse, 20 ms pulse width). We plated the cells immediately into 6- or 24-well plates after electroporation and cultured them for 72 hours before FACS sorting. We changed the media twice in the 8–24 hours post-electroporation and once daily thereafter. These frequent media changes are critical for promoting cell survival following the stressful electroporation procedure. Each electroporation experiment included several controls, including untransfected, mCherry plasmid only, ABEmax-P2A-GFP plasmid only, and GFPmax reporter plasmid (Lonza). For the controls, we added an empty plasmid vector to the reactions to ensure that the same amount of DNA was loaded into both the samples and the controls.

## Fluorescence-Automated Cell Sorting (FACS)

We detached the cells from the culture dish at 72 hours post-transfection via TrypLE Express without phenol red (Thermo Fisher: 12604013). We centrifuged these cells at 300 × g for four minutes and resuspend them Thein 0.5 mM EDTA in PBS (Sigma: E8008) to reduce cell clumping. We then sorted the cell mixture for mCherry + GFP + double-positive cells via a conservative gating strategy based on the compensation controls (mCherry only, GFP only, and untransfected). We sorted the cells via the BD FACSAria™ III Cell Sorter (Becton Dickinson, Franklin Lakes, NJ) with a 100 µm nozzle and the "purity" collection setting. We extracted DNA from the sorted cell population immediately after sorting via the QIAGEN DNeasy Blood & Tissue Kit (QIAGEN Sciences: #69504, Germantown, MD).

## EditR Quantification and Statistical Methods

First, we validated the quality of the DNA harvested from previously sorted cells by measuring the 260/280 (1.8–2.0) and 260/230 ratios (2.0–2.2) with a Nanodrop (Thermo Fisher). Next, we performed two polymerase chain reactions (PCRs). The first was a long-range PCR for exons 13–21 in *PKD1*, and the second was a short-range PCR directed against the *PKD1*$^{Q2556X}$ region. This additional long-range PCR step is necessary because *PKD1* has several pseudogenes, and this protocol ensures that only the true *PKD1* coding sequence is amplified in the second PCR. The full PCR protocol is described in detail in **Supplementary Note 3.** We sent the final short-range products for Sanger sequencing (Eton Biosciences, San Diego, CA). Next, we used editR software (University of Minnesota) to quantify the editing efficiency based on the Sanger sequencing plots (26). EditR uses a p-value of 0.01 and designates significantly different base pairs with an orange color (compared with untransfected controls).

## Declarations

**Ethics approval and consent to participate:** Not applicable

**Consent for publication:** Not applicable

**Availability of data and materials:** The data underlying this article are available in the article and in its online supplementary material. Any additional raw data not contained in the supplementary material will be shared upon reasonable request to the corresponding author.

**Competing interests:** DSM and NSJ are employees of Nephrogen Inc. GMC and VB receive compensation from Nephrogen.

**Funding:** This work was supported by the Stanford Maternal and Child Health Research Institute, the Stanford SPARK Program for Translational Research, and the National Institutes of Health (1R41DK138689 to D.S.M).

**Authors' contributions:** DSM conceptualized the project, conducted all the *in vitro* experiments, provided funding for the research, and wrote the initial manuscript draft. DWW and NSJ created the software for the editABLE website and revised the manuscript. VC, JNK, SA, and GMC conceptualized the project and revised the manuscript. VB conceptualized the project, analyzed the data, and provided funding for the research. All authors read and approved the final manuscript.

**Acknowledgements:** We thank Lily Margaret Thompson and Fernando Gomez Garcia for their helpful discussions. The cell sorting/flow cytometry analysis for this project was performed via instruments at the Stanford Shared FACS Facility.

## Figures

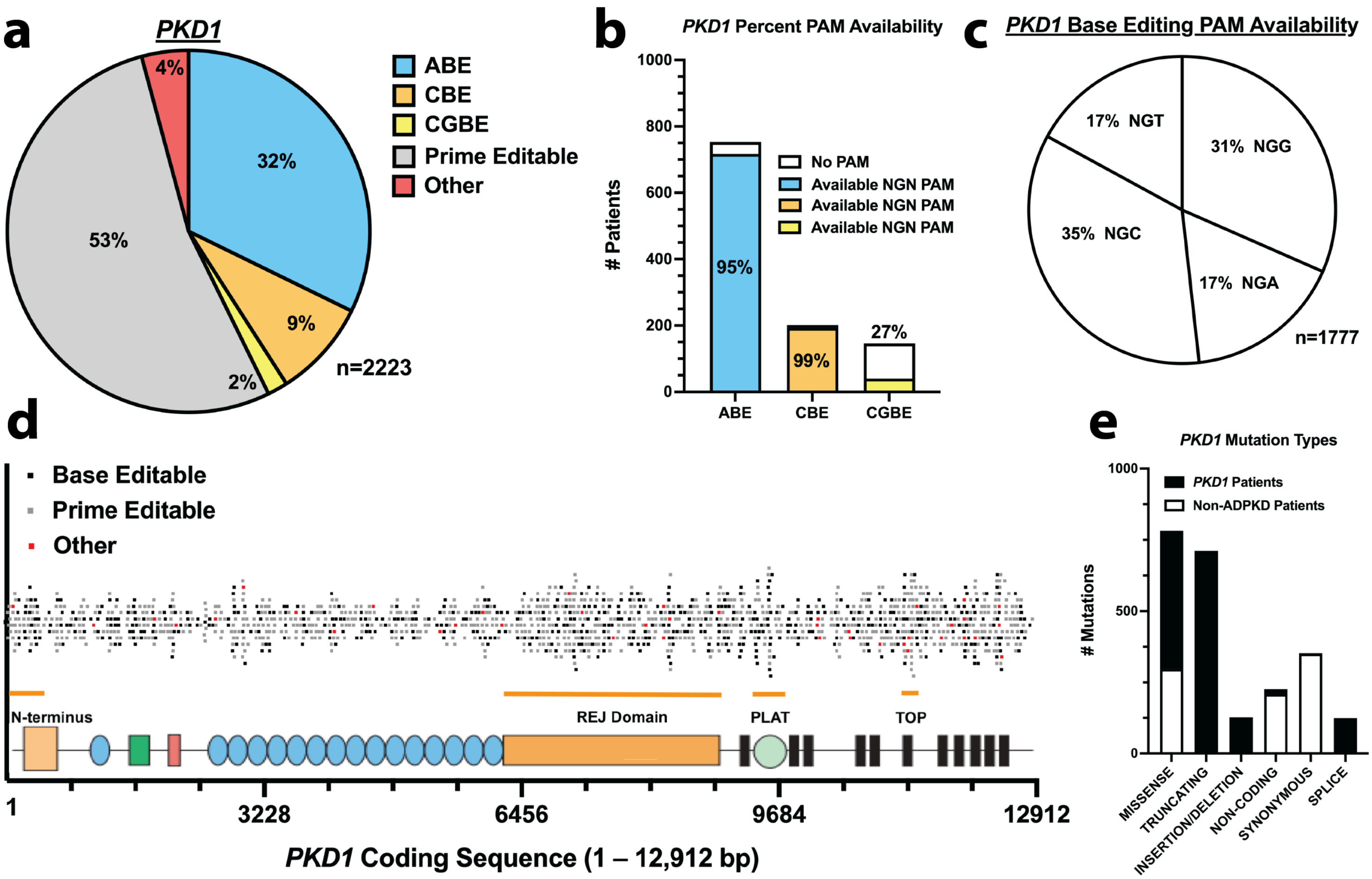

Figure 1

**EditABLE shows that >95% of *PKD1* patients carry mutations amenable to base or prime editing.** Using a Python script, we applied the editABLE algorithm to the Mayo Clinic ADPKD Mutation Database. All *PKD1* variants from patients without ADPKD in the Mayo Clinic database were excluded from the analysis except for part **(e)**.**(a)** Pie chart showing the percentage of *PKD1* patients amenable to each base or prime editing technology. Patients were classified as amenable to base editing (ABE, CBE, or CGBE) if their mutation possessed an NGN PAM sequence that positioned the substitution mutation 12–17 bp upstream of the PAM in the 5' direction. **(b)** Bar graph showing the percentage of patients who possess the appropriate substitution mutations for base editing but do not have NGN PAM sequences available in the optimal editing window. **(c)** Pie chart showing which third nucleotides are most common in NGN PAM sequences. NGG is the most efficient for base editing (6). The data from **(a)** are plotted as a dot plot against the **(d)** *PKD1* coding region. Each dot represents an individual mutation from the Mayo Clinic ADPKD Mutation Database. All mutations from the Mayo database are shown except for large deletion or duplication mutations spanning multiple exons. Note that the *PKD1* gene is located on the 3' strand, so the sequence is shown in reverse. **(e)**Bar graph showing a breakdown of mutation types in the ADPKD mutation database with the percentage of pathogenic mutations vs. non-ADPKD mutations for each mutation type. ABE, adenine base editor; CBE, cytosine base editor; CGBE, transversion base editor; PAM, protospacer-adjacent-motif; REJ, receptor for egg jelly; TOP, terminal oligo pyrimidine; PLAT, polycystin-1 lipoxygenase alpha-toxin.

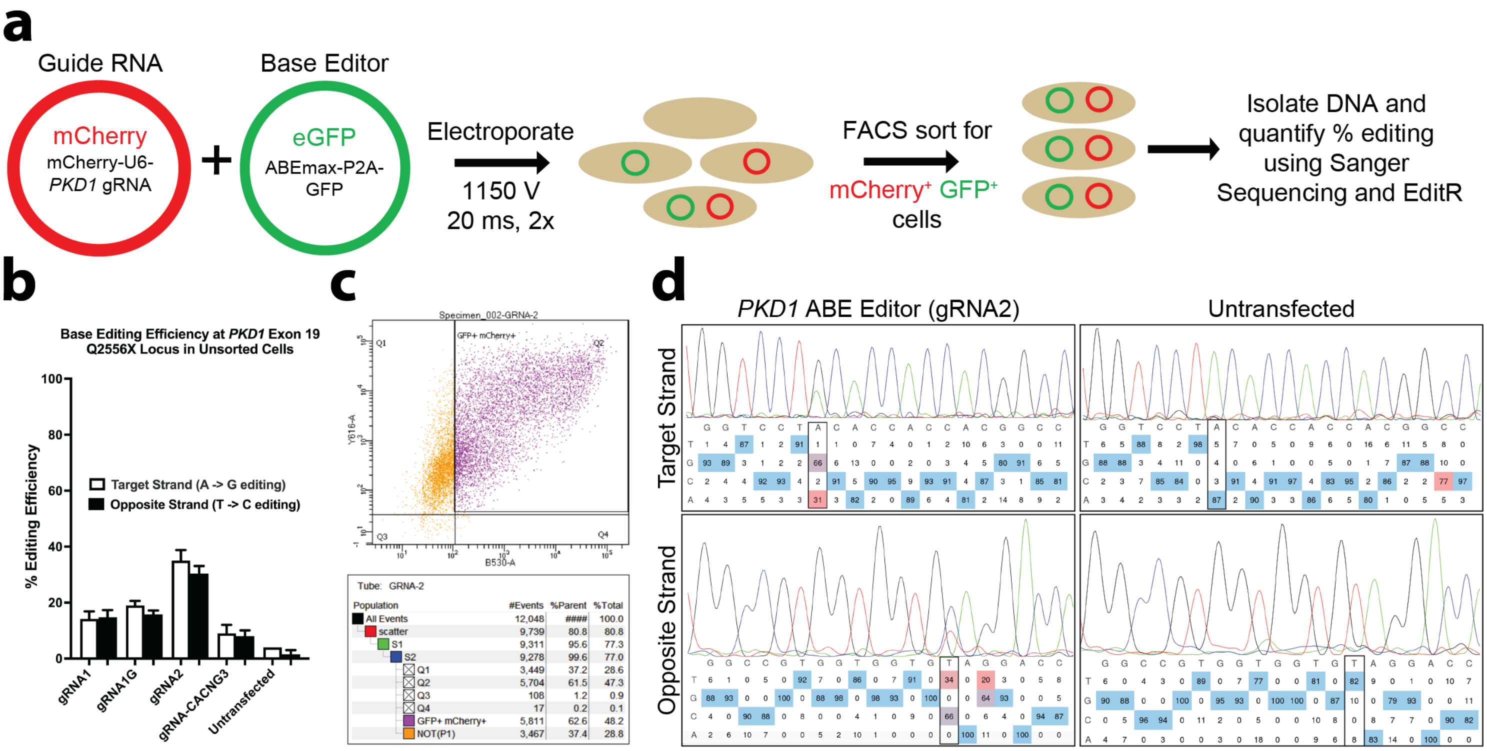

### Figure 2

**EditABLE identifies high efficiency base editors for correcting pathogenic *PKD1* mutations *in vitro*. (a)** Schematic overview of the experimental protocol for validating base-editing gRNAs identified through the editABLE algorithm. Editing efficiency was quantified in both unsorted and sorted cells for three ABE guide RNAs: gRNA1, gRNA1G (a version of gRNA1 with the first base pair modified to guanine), and gRNA2. **(b)** Bar graph showing editing efficiency in unsorted human renal epithelial cells, WT 9-12 (see Methods for details) on both the target strand and reverse strand. DNA was extracted 72 hours posttransfection, amplified via PCR, and analyzed for editing efficiency via Sanger sequencing and EditR analysis. **(c)** FACS plot showing 62.6% mCherry+ GFP+ double positive cells. **(d)** EditR plots for the most efficient guide RNA (gRNA2) showing statistically significant 66% A->G editing efficiency on both the forward and reverse strands. Untransfected controls are shown on the right and no variations in the base pair sequence according to EditR analysis are statistically significant. gRNA, guide RNA; ABE, adenine base editor; GFP, green fluorescent protein; fluorescence-activated cell sorting, FACS. *, p-value < 0.01.

## Supplementary Files

This is a list of supplementary files associated with this preprint. Click to download.

- EditABLEManuscriptSupplementV5.docx
- SuppTables.xlsx
